# Global profiling of O-GlcNAcylated and/or phosphorylated proteins in hepatoblastoma

**DOI:** 10.1038/s41392-019-0067-4

**Published:** 2019-10-11

**Authors:** Hang Song, Ji Ma, Zhixuan Bian, Shuhua Chen, Jiabei Zhu, Jing Wang, Nan Huang, Minzhi Yin, Fenyong Sun, Min Xu, Qiuhui Pan

**Affiliations:** 1 Department of Laboratory Medicine, Shanghai Children’s Medical Center, School of Medicine, Shanghai Jiaotong University, 200127 Shanghai, China; 2 Department of Laboratory Medicine, Yunfu People’s Hospital, 527300 Guangdong, China; 3 Department of Surgery, Shanghai Children’s Medical Center, School of Medicine, Shanghai Jiaotong University, 200127 Shanghai, China; 4 Department of Laboratory Medicine, Shanghai Tenth People’s Hospital of Tongji University, 200072 Shanghai, China; 5 Department of Pathology, Shanghai Children’s Medical Center, School of Medicine, Shanghai Jiaotong University, 200127 Shanghai, China

**Keywords:** Epigenetics, Epigenetics, Molecular medicine

## Abstract

O-linked-β-N-acetylglucosamine (O-GlcNAc) glycosylation (O-GlcNAcylation) and phosphorylation are critical posttranslational modifications that are involved in regulating the functions of proteins involved in tumorigenesis and the development of various solid tumors. However, a detailed characterization of the patterns of these modifications at the peptide or protein level in hepatoblastoma (HB), a highly malignant primary hepatic tumor with an extremely low incidence in children, has not been performed. Here, we examined O-GlcNAc-modified or phospho-modified peptides and proteins in HB through quantitative proteomic analysis of HB tissues and paired normal liver tissues. Our results identified 114 O-GlcNAcylated peptides belonging to 78 proteins and 3494 phosphorylated peptides in 2088 proteins. Interestingly, 41 proteins were modified by both O-GlcNAcylation and phosphorylation. These proteins are involved in multiple molecular and cellular processes, including chromatin remodeling, transcription, translation, transportation, and organelle organization. In addition, we verified the accuracy of the proteomics results and found a competitive inhibitory effect between O-GlcNAcylation and phosphorylation of HSPB1. Further, O-GlcNAcylation modification of HSPB1 promoted proliferation and enhanced the chemotherapeutic resistance of HB cell lines in vitro. Collectively, our research suggests that O-GlcNAc-modified and/or phospho-modified proteins may play a crucial role in the pathogenesis of HB.

## Introduction

Hepatoblastoma (HB), which originates from the abnormal development of pluripotent stem cells or hepatic progenitor cells, is a highly malignant embryonic hepatocellular carcinoma in children. According to the National Cancer Institute, the occurrence of HB is extremely rare, with approximately 1.5 cases per million individuals each year.^1^ Despite the improvements and advancements in surgical management, medication, and postoperative chemoradiotherapy for HB, the majority of HB patients still have an unfavorable prognosis with a high risk of early death due to difficulties in diagnosis and the high frequency of metastasis and recurrence.^2,3^ Therefore, it is critical to fully understand the molecular mechanism and pathogenesis of HB, which would be helpful to improve the early diagnosis, treatment, and prognosis of HB patients.

Posttranslational modifications (PTMs) of proteins are important mechanisms for modulating protein function and increasing the proteome complexity in organisms.^4^ O-linked-β-N-acetylglucosamine (O-GlcNAc) glycosylation (O-GlcNAcylation) involves a single N-acetylglucosamine molecule being transferred to or removed from serine (Ser) and/or threonine (Thr) residues of proteins by O-GlcNAc transferase (OGT) or O-GlcNAcase, respectively.^5^ O-GlcNAcylation is a widespread, dynamic and reversible PTM that is involved in the regulation of diverse cellular regulatory processes, including protein–protein interactions, cellular signal transduction, epigenetic reprogramming, and metabolic regulation.^6–8^ Moreover, numerous O-GlcNAcylated proteins have been found to be associated with the progression and development of various kinds of cancers. For example, O-GlcNAcylation of the ribosomal protein RACK1 at Ser122 resulted in a dramatic decrease in the malignancy of hepatocellular carcinoma cells in vitro and in vivo.^9^

Phosphorylation of proteins by protein kinases has been demonstrated to play a regulatory role in many biological functions, such as signal transduction, the cell cycle, cell metabolism, and protein subcellular localization.^10–13^ For instance, a previous study showed that p-38 mitogen-activated protein kinases (MAPKs), extracellular signal-related kinase (ERK 1/2), and Akt were phosphorylated and involved in apoptosis and cell cycle arrest in HB cells.^14^ Interestingly, Ser/Thr sites in proteins can be phosphorylated, as well as O-GlcNAcylated, suggesting the possibility of the dynamic and extensive crosstalk between O-GlcNAcylation and phosphorylation.^15^ Indeed, accumulating evidence has shown that the majority of phosphorylated proteins are also O-GlcNAcylated.^16,17^ Further, an interaction between O-GlcNAcylation and phosphorylation was demonstrated in the mouse synaptosome and in the Arabidopsis model plant system.^18,19^ Previously, we elucidated that global O-GlcNAcylation expression levels were increased in liver cancer cell lines and tissues, which simulated the tumorigenesis of liver cancer.^17^ However, whether O-GlcNAcylation is involved in the pathways driving HB tumor progression and development and whether key regulatory proteins are O-GlcNAcylated and phosphorylated have not been examined.

In this study, we performed large-scale identification of O-GlcNAcylated and phosphorylated proteins in HB and normal liver tissues and examined the related physiological processes. Our data shed light on the potential key regulatory proteins in HB and helped provide a foundation for the functional study of O-GlcNAcylated or phosphorylated proteins in HB.

## Results

### O-GlcNAcylation and OGT levels were upregulated in HB tissues

**Fig. 1:**
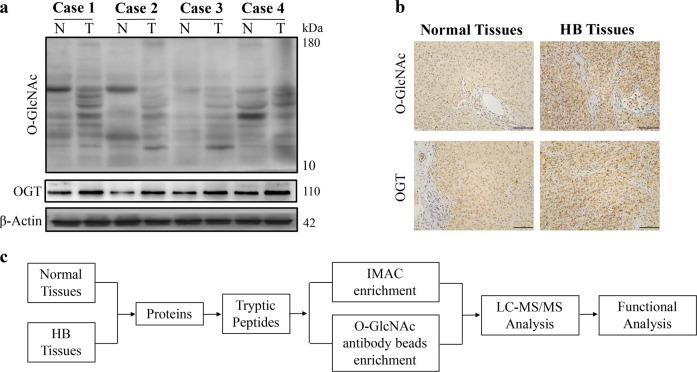
O-GlcNAcylation and phosphorylation in HB tissues. The expression levels of total O-GlcNAcylation and OGT in HB tissues and normal tissues were measured by using western blot (**a**) and immunohistochemical analyses (**b**, scale bars, 100 μm). **c** Flowchart for the enrichment and identification of O-GlcNAcylated and phosphorylated peptides by proteomic analysis. All experiments were repeated three times

Here, we sought to determine whether O-GlcNAcylation played a critical role in the development of HB. Western blot and immunohistochemical analyses were performed in 4 paired HB tissues and matched noncancerous normal tissues. The results showed that the levels of both total O-GlcNAcylation and O-GlcNAc transferase (OGT) were elevated in all HB tissues compared with those in the matched normal tissues (Fig. 1a, b).

### O-GlcNAcylation and phosphorylation in the proteome of HB and normal tissues

The high levels of O-GlcNAc modification in HB tissues suggested that O-GlcNAcylation might be associated with the pathogenesis of HB. Previous studies have shown that O-GlcNAcylation and phosphorylation can target the same Ser/Thr site(s) or different Ser/Thr sites.^15^ Hence, it is essential to identify O-GlcNAcylated and/or phosphorylated sites and proteins and determine their potential regulatory role in HB. Thus, we employed the workflow of PTM Bio (Zhejiang, China) to identify and compare O-GlcNAcylated and phosphorylated proteins in the abovementioned 4 pairs of HB and normal matched tissues (Fig. 1c). Proteins were extracted from tissues, and O-GlcNAc-modified or phospho-modified peptides were enriched using O-GlcNAc antibody beads and Immobilized metal affinity chromatography (IMAC). Peptide samples were processed by tandem mass spectrometry (MS/MS), and MaxQuant was used to analyze the MS/MS data. The reproducibility of quantitative proteomic measurements among the four patient samples was precisely evaluated, and the results are shown in Supplementary Table S1 and Supplementary Data S1 and Supplementary Data S2.

The data analysis identified 114 differentially expressed O-GlcNAcylated sites mapping to 78 proteins from both HB tissues and normal tissues and 3494 unique phosphorylation sites in 2088 proteins (Table 1). Details of the modified sites and peptides are listed in Supplementary Data S3 and Supplementary Data S4.

**Table 1:** MS/MS spectrum database search analysis summary

Modification	O-GlcNAcylation	Phosphorylation
Total spectrums	39,635	114,007
Matched spectrum	2046 (5.2%)	23,624 (20.7%)
Peptides	1223	6254
Modified peptides	114	3494
Identified proteins	78	2088 (1894)
Identified sites	141	4546 (3372)

### Overview of the identified O-GlcNAcylated and phosphorylated peptides and proteins

**Fig. 2:**
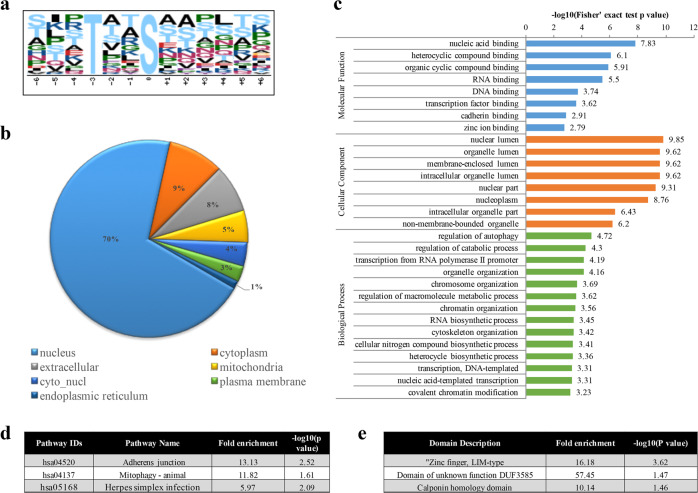
Summary of identified O-GlcNAcylated proteins and peptides. **a** Motif analysis of O-GlcNAcylated sites identified in this study. **b** Subcellular localization of identified O-GlcNAcylated proteins. **c** Gene Ontology (GO) analysis of O-GlcNAcylated proteins. **d** KEGG pathway analysis of all O-GlcNAcylated proteins. **e** Protein domain enrichment analysis of O-GlcNAcylated proteins

To more closely examine the characteristics of the modified sites, we performed motif analysis on the flanking sequences of these sites. In O-GlcNAcylated proteins, there was a preference for threonine residues at the -3 position (Fig. 2a). In phosphorylated proteins,…R..SP.P…,……SP…R.,…R..SP….. and……SP.R… were characterized as the top four upregulated motifs for phosphorylation sites (Fig. S1a). Details of the motifs in phosphorylated peptides are shown in Supplementary Data S5.

WoLF PSORT was used to predict the subcellular localization of the identified O-GlcNAcylated or phosphorylated proteins. The majority of the detected O-GlcNAcylated or phosphorylated proteins were predicted to accumulate in the nucleus, cytoplasm, extracellular space, mitochondria, and endoplasmic reticulum. A few phospho-modified proteins tended to be localized in the peroxisome, cytoskeleton, and Golgi apparatus (Fig. 2b, Fig. S1b).

Gene Ontology (GO) enrichment analysis was performed to elucidate the cellular components, molecular functions and biological processes of O-GlcNAcylated or phosphorylated proteins. The results indicated that O-GlcNAc-modified and phospho-modified proteins are components of the nuclear and organelle lumen and the cell junctions, respectively. In addition, O-GlcNAcylated proteins are involved in nucleic acid binding and are enriched in the terms regulation of autophagy, catabolic process, and transcription from RNA polymerase II (Fig. 2c). Unlike O-GlcNAcylated proteins, phospho-modified proteins were enriched in RNA binding and associated with mRNA processing and RNA splicing (Fig. S1c).

KEGG pathway annotation revealed that O-GlcNAcylated proteins were related to adherens junction and mitophagy (Fig. 2d), whereas phosphorylated proteins were associated with multiple signaling pathways, including the spliceosome, tight junction and glucagon pathways (Fig. S1d). Protein domain enrichment analysis showed that zinc finger, LIM-type, domain of unknown function DUF3585 and calponin homology domains were enriched in O-GlcNAcylated proteins (Fig. 2e). Phosphorylated proteins were enriched in nucleotide-binding alpha-beta plait domains, RNA recognition motif domains and pleckstrin homology domains (Fig. S1e).

### Functions of O-GlcNAcylated proteins

**Fig. 3:**
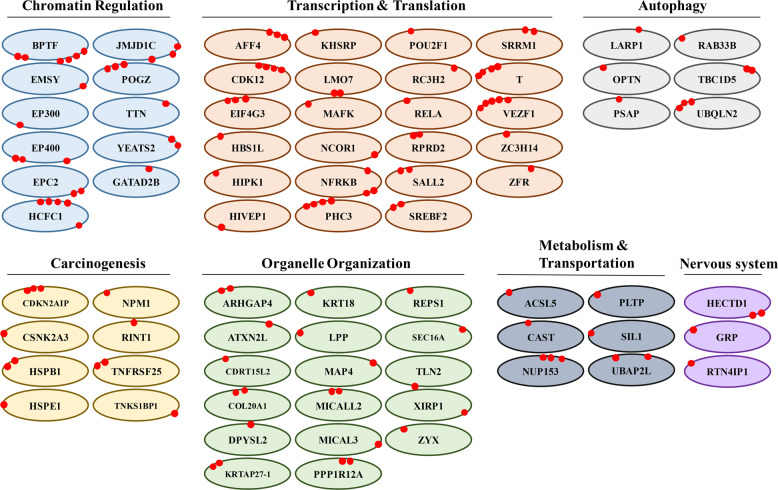
Functions of O-GlcNAcylated proteins. Proteins with O-GlcNAcylation modification were classified into several groups according to their known functions. Each red dot represents an O-GlcNAc-modified site

While numerous studies have elucidated the functions of phosphorylated proteins, little is known about the role of O-GlcNAc-modified proteins. As illustrated in Fig. 3, O-GlcNAcylated proteins identified in this study were associated with multiple biological processes. Many of the identified O-GlcNAcylated proteins, including histone acetyltransferase p300 (EP300) and the nucleosome-remodeling factor subunit BPTF, were related to chromatin remodeling. The majority of these proteins are transcription and translation modifiers that participate in mRNA elongation (AFF4^20^ and CDK12^21^), degradation (KHSRP^22^), splicing (SRRM1^23^) and deadenylation (RC3H2^24^). These proteins are also involved in organelle organization—for example, the critical components of transitional ER organization, SEC16A^25^ and KRT18, which play a key role in the reorganization of intermediate filaments.^26^ In addition, several O-GlcNAcylated proteins were linked to the regulation of autophagy and carcinogenesis. A few O-GlcNAc-modified proteins were associated with metabolism, transportation and the nervous system.

### Crosstalk between O-GlcNAcylation and phosphorylation

**Fig. 4:**
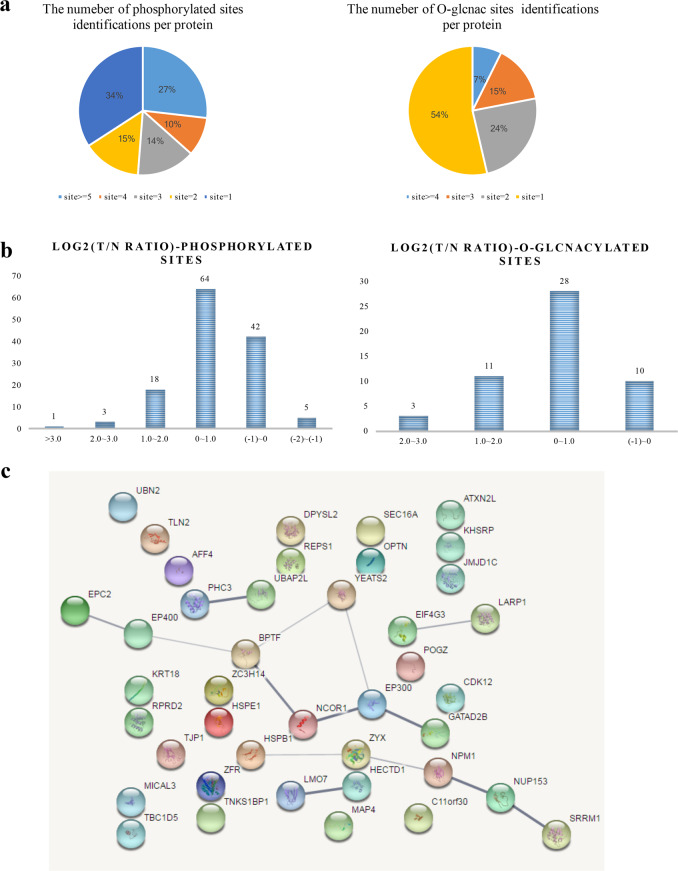
Overview of proteins and peptides modified by both O-GlcNAcylation and phosphorylation. **a** Numerical distribution of modified sites. **b** Quantitative distribution of modified sites. **c** Protein–protein interaction network of proteins modified by both O-GlcNAcylation and phosphorylation

The distribution of the number of modified sites identified per protein is shown in Fig. 4a, b. The majority of the O-GlcNAcylated or phosphorylated proteins had only 1 modified site. In addition, log2 transformation was performed to quantify the modification sites on the modified proteins, and most of the ratios (T/N) were between 0 and 1. In addition, our data revealed that 52% of the identified O-GlcNAcylated proteins were also modified by phosphorylation and that these proteins were related to chromatin regulation, transcription, translation, transportation, and organelle organization. To more closely examine the interaction networks among these proteins, protein complex enrichment analysis was performed. These results showed that some of the proteins modified by both O-GlcNAcylation and phosphorylation could interact with each other (Fig. 4c) and thus might play a synergistic role in cellular signal transduction.

### Verification of the MS data

**Fig. 5:**
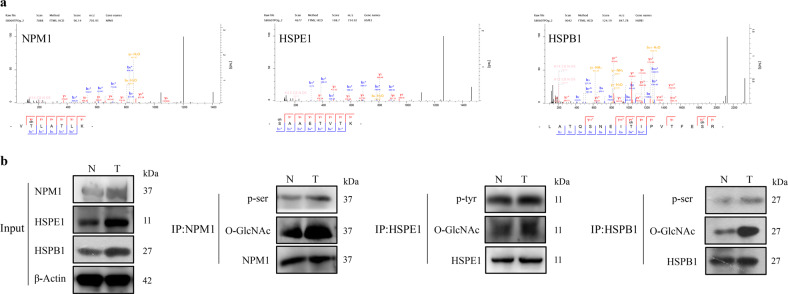
NPM1, HSPE1, and HSPB1 were both O-GlcNAc-modified and phospho-modified. **a** ETD mass spectra of peptides derived from NPM1, HSPE1, and HSPB1. **b** The O-GlcNAcylation and phosphorylation levels of endogenous NPM1, HSPE1, and HSPB1 were measured in HB tissues and normal tissues by immunoprecipitation and western blot analysis. All experiments were repeated three times

Among the proteins modified by both O-GlcNAcylation and phosphorylation, NPM1, HSPE1, and HSPB1 attracted our attention, as these proteins are involved in tumorigenesis.^27–29^ We further verified the accuracy of the MS data by performing immunoprecipitation and western blot analysis in HB tissues. These results showed that NPM1, HSPE1, and HSPB1 were modified by both O-GlcNAcylation and phosphorylation, and the levels of NPM1, HSPE1, and HSPB1 O-GlcNAcylation and phosphorylation were significantly higher in HB tissues than in normal tissues (Fig. 5a, b).

### HSPB1 is O-GlcNAc-modified and associated with the survival and chemotherapy resistance of HB cell lines

**Fig. 6:**
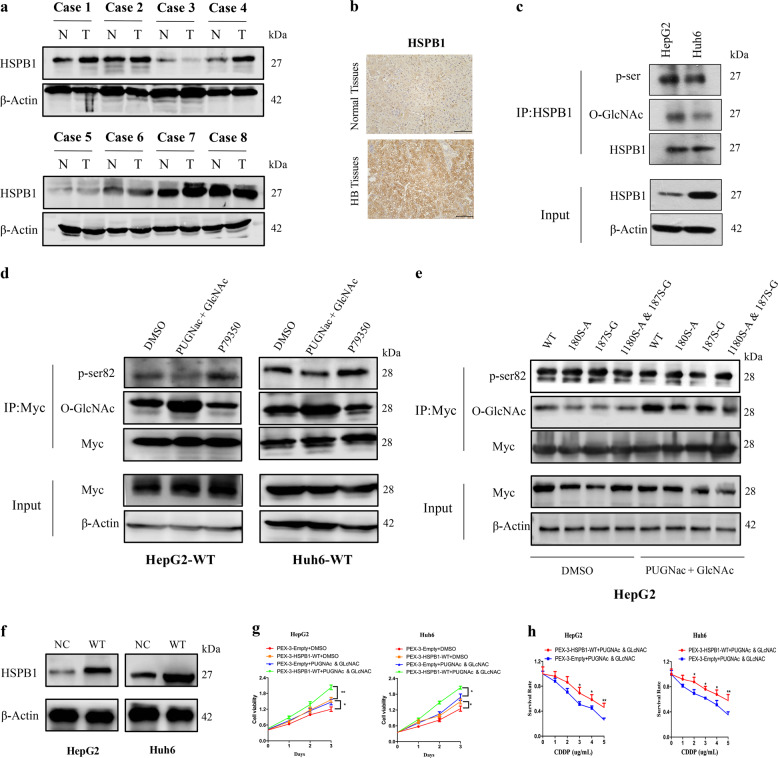
HSPB1 is O-GlcNAc-modified and involved in the chemotherapy resistance of HB cell lines. **a**, **b** Western blotting and immunohistochemistry were performed to determine the expression pattern of HSPB1 in HB tissues and normal tissues. **c** O-GlcNAcylation and phosphorylation modifications of HSPB1 were detected in HepG2 and Huh6 cells. **d** Cells were treated with PUGNAc (25 mM) and GLcNAC (4 mM) for 48 h or with P79350 (50 nM) for 0.5 h, and cell lysates were immunoprecipitated with an anti-Myc antibody, followed by western blotting with either an anti-MYC antibody, anti-O-GlcNAc antibody, or anti-phospho-HSPB1 (Ser82) antibody. **e** Cells transfected with Myc-HSPB1-WT or Myc-HSPB1-mutant plasmids were exposed to P79350 (50 nM) or to PUGNAc (25 mM) and GLcNAC (4 mM) for 0.5 h or 48 h, respectively. Cell lysates were immunoprecipitated with an anti-Myc antibody, followed by western blot analysis. **f** Cells were transfected with Myc-HSPB1-WT plasmid, and western blotting was conducted to evaluate the transfection efficiency 48 h later. **g** CCK-8 proliferation assays were applied to assess the effect of HSPB1 O-GlcNAcylation on the survival of HB cells. **h** CCK-8 proliferation assays were performed to evaluate the effect of HSPB1 O-GlcNAcylation on cisplatin tolerance. The error bars indicate the SD, *p < 0.05; **p < 0.01. All experiments were repeated three times

Previous studies demonstrated that heat shock protein beta-1 (HSPB1) is abnormally expressed in various human cancers, including glioma,^30^ hepatocellular carcinoma,^31^ non-small-cell lung cancer^32^, and breast cancer.^33^ However, the expression and function of HSPB1 in HB are not known. Western blotting and immunohistochemistry revealed higher expression of HSPB1 in HB tissues than in the matched normal tissues (Fig. 6a, b). Consistent with the results in HB tissues, O-GlcNAcylation and phosphorylation modification of HSPB1 were observed in the HB cell lines HepG2 and Huh6 (Fig. 6c). It has been reported that phosphorylation of the serine residue at position 82 contributes to the diverse functional roles of HSPB1.^34–36^ We next examined whether there was potential competition between O-GlcNAcylation and phosphorylation of Ser82 in HSPB1. HB cells transfected with Myc-HSPB1-WT were treated with PUGNAc (25 mM) and GLcNAC (4 mM) or with P79350 (50 nM) to increase the levels of O-GlcNAcylation or phosphorylation of HSPB1 Ser82, respectively. Interestingly, the data demonstrated that the levels of HSPB1 O-GlcNAcylation and phosphorylation in HB cells were dynamically changed. As shown in Fig. 6d, we observed increased levels of O-GlcNAc-modified HSPB1 after stimulation toward O-GlcNAcylation, with a parallel decrease in HSPB1 phosphorylation (Ser82) in both HepG2 and Huh6 cells.

The MS data suggested that 180S and 187T in HSPB1 were modified by O-GlcNAcylation. To confirm whether these PTMs occurred at 180S and/or 187T in HSPB1, we generated a panel of plasmids expressing point mutations of HSPB1 (Myc-HSPB1-T180A, Myc-HSPB1-S187G, and Myc-HSPB1-T180A/S187G). We transfected cells with the mutant plasmids and exposed the cells to P79350 (50 nM) or to PUGNAc (25 mM) and GLcNAC (4 mM) for 0.5 h or 48 h, respectively. Myc immunoprecipitation was performed, followed by western blotting. While a strong increase in O-GlcNAcylation of wild-type HSPB1 was observed, the T180A, S187G, and T180A/S187G mutants showed relatively reduced levels of O-GlcNAcylation, suggesting that HSPB1 might be O-GlcNAcylated at S180 and T187. Hence, we sought to determine whether the crosstalk between the O-GlcNAcylation and phosphorylation of Ser82 was mediated by O-GlcNAcylation of Ser180 and/or Tyr187. However, mutation of Ser180 and/or Tyr187 had little effect on the phosphorylation of Ser82 in HB cells with or without stimulation toward O-GlcNAcylation (Fig. 6e), indicating that other sites in HSPB1 are O-GlcNAc-modified. Notably, the proliferation experiment showed that overexpressing HSPB1 or increasing the O-GlcNAcylation of HSPB1 alone increased cell proliferation, whereas advanced O-GlcNAcylation of HSPB1 displayed a superior ability for cell multiplication in tumor progression (Fig. 6f, g). In addition, O-GlcNAcylation of HSPB1 stimulated the tolerance of HB cells to cisplatin in vitro. These results further suggested that HSPB1 might serve as a therapeutic target in HB (Fig. 6h).

## Discussion

Multiple proteomic studies have shown that O-GlcNAcylation and phosphorylation modifications serve as key regulators of various physiological functions.^6–8,10–13^ Previous research has identified and characterized a large set of O-GlcNAcylated or phosphorylated proteins in murine synapses and Arabidopsis.^18,19^ In addition, the role of O-GlcNAcylation and phosphorylation has been examined for specific proteins. However, a comprehensive investigation and survey of both O-GlcNAcylation and phosphorylation in human tumors has not been conducted, especially in HB, a rare disease with a largely unknown pathogenesis.^1–3^ Our study is the first to examine the O-GlcNAcylated and phosphorylated proteins in HB. GO and KEGG analyses uncovered multiple functions for these proteins in various physiological and biochemical processes. These data provide a broad basis and foundation for further research on the functional consequence of the O-GlcNAcylation or phosphorylation of specific proteins in HB and other kinds of human tumors.

Interestingly, our results demonstrated that the sequence preference of the identified O-GlcNAc-modified or phospho-modified sites was dissimilar to that found in other diseases and plants, indicating that the enzymatic characteristics of OGTs and protein kinases are distinct in different microenvironments. The majority of the modified proteins in our project were predicted to localize in the nucleus and cytoplasm, and functional analysis suggested that these proteins were involved in mRNA transcription, translation and chromatin remodeling. These data demonstrated that the locations and functions of the identified O-GlcNAcylated or phosphorylated proteins were similar to those of proteins in plants,^19^ revealing the evolutionary conservation of these proteins.

Our data revealed 114 differentially expressed O-GlcNAc-modified sites in 78 proteins and 3494 phosphorylation sites in 2088 proteins. These numbers are slightly lower than those in the dataset of modified proteins in other tissues.^18,19^ However, the results of GO and KEGG analyses provided adequate evidence that O-GlcNAcylated or phosphorylated proteins with important biological roles were identified in our project. In addition, we verified the data for a subset of the identified proteins in HB tissues and cell lines. Mutagenesis studies confirmed that the O-GlcNAc-modified sites in HSPB1 were consistent with the sites identified via proteomic analysis and that there was crosstalk between the O-GlcNAcylation and phosphorylation of HSPB1. These findings provided evidence for the accuracy of our data.

Several considerations should be noted regarding our study. Notably, our initial analysis failed to identify β-catenin, which has been shown to be O-GlcNAc-modified or phosphorylated in various kinds of cancers.^37–40^ Further, the number of samples in the present study is small. Therefore, a larger sample of HB tissues and matched normal tissues is required to improve the precision and accuracy of these results. However, our proteomics data provide valuable preliminary information for future functional studies.

Recent reports have shown that autophagy plays a critical role in tumor development and progression by stimulating progression through supplying sufficient amounts of essential nutrients or eliminating damaged or mutated macromolecules in cancer cells.^41,42^ Notably, several O-GlcNAc-modified autophagy proteins were identified in our dataset, including optineurin (OPTN), which is an autophagy modifier in the MAP1 LC3 family.^43^ In addition, our results showed the O-GlcNAcylation of several tumor-associated proteins, such as CDKN2AIP^44^ (a key regulator of DNA damage and repair), CSNK2A3^45^ (a tumor growth-promoting factor) and RINT1^46^ (which functions as a modifier in cell cycle checkpoint control). Our results suggested that these proteins might be involved in the development and progression of HB, and future studies should examine their function, because they may serve as molecular targets in HB.

Given that O-GlcNAcylation and phosphorylation may occur on nearby or on the same Ser/Thr residues, we analyzed the protein interaction network of the O-GlcNAcylated and phosphorylated proteins via the STRING database. We found that proteins that were both O-GlcNAcylated and phosphorylated were involved in critical physiological processes, such as chromatin regulation, transcription and translation. In addition, several of the proteins that were both O-GlcNAcylated and phosphorylated were predicted to interact with each other, suggesting the important role of O-GlcNAcylation and phosphorylation modifications in the regulation of HB.

Taken together, our data provide comprehensive information on the O-GlcNAc-modified and/or phospho-modified peptides and proteins in HB. Notably, the locations and functions of these proteins were evolutionarily conserved. Functional prediction analysis indicated that these modified proteins might serve as critical regulators of diverse cellular and molecular processes. These findings will help further our understanding of the biological function of protein O-GlcNAcylation and phosphorylation in HB. Furthermore, our data lay a foundation for research on the crosstalk between protein O-GlcNAcylation and phosphorylation in other diseases.

## Materials and methods

### Drugs and reagents

Doxorubicin and cisplatin were acquired from Selleck Chemical (TX, USA), PUGNAc and GlcNAc were purchased from Sigma-Aldrich (MO, USA), and P79350 was obtained from Invitrogen (CA, USA). All other reagents and chemicals, unless otherwise stated, were obtained from Beyotime (Jiangsu, China).

### Specimens and immunohistochemical staining

Four pairs of cancerous tissues and matched noncancerous normal liver tissues were collected from HB patients who underwent surgery at the Department of Hepatobiliary Surgery of the Shanghai Children’s Medical Center. Patients did not receive chemotherapy or radiotherapy before admission. This study was approved by the Ethics Committee of the Shanghai Children’s Medical Center, and written informed consent was obtained from each patient. The detailed information of these patients is listed in Supplementary Table S2.

Immunohistochemical staining was performed as previously described.^47^ The primary antibodies used were anti-OGT (D1D8Q) rabbit mAb (#24083, Cell Signaling Technology, MA, USA), anti-O-GlcNAc mouse mAb (PTM-952, PTM Bio, Zhejiang, China), and anti-HSPB1 (D6W5V) rabbit mAb (#95357, Cell Signaling Technology, MA, USA).

### Cell culture

The human HepG2 and Huh6 HB cell lines were purchased from the Cell Bank of the Chinese Academy of Sciences (Shanghai, China). Both cell lines have been authenticated by STR genotyping. HepG2 and Huh6 cells were cultured in minimum essential medium (MEM, HyClone, UT, USA) and Dulbecco’s modified Eagle’s medium (DMEM, HyClone, UT, USA), respectively, supplemented with 10% fetal bovine serum (Gibco, CA, USA), 100 U/ml streptomycin (Invitrogen, CA, USA) and 100 U/ml penicillin (Invitrogen, CA, USA) at 37 °C in a humidified atmosphere with 5% CO_2_.

### Western blot analysis

Tissues and cells were lysed in western lysis buffer (Beyotime, Jiangsu, China) for 30 min on ice, followed by centrifugation at 12,000×*g* for 15 min. Protein samples (30 μg) were separated on SDS–PAGE gels and transferred to PVDF membranes (GE Healthcare, Buckinghamshire, UK). Membranes were blocked with 5% BSA at room temperature for 1 h, followed by overnight incubation with primary antibodies at 4 °C. Membranes were washed with PBST three times, followed by incubation with the appropriate HRP-conjugated secondary antibodies (Santa Cruz, CA, USA) at room temperature for 1 h. Bands were visualized using a SuperSignal West Femto kit (Pierce, IL, USA). GAPDH was used as the loading control. The primary antibodies used were anti-OGT (D1D8Q) rabbit mAb (#24083, Cell Signaling Technology, MA, USA), anti-O-GlcNAc mouse mAb (PTM-952, PTM Bio, Zhejiang, China), anti-HSPB1 (D6W5V) rabbit mAb (#95357, Cell Signaling Technology, MA, USA), anti-Phospho-HSPB1 (Ser82) (D1H2F6) rabbit mAb (#9709, Cell Signaling Technology, MA, USA), anti-NPM1 rabbit pAb (10306-1-AP, Proteintech, IL, USA), anti-HSPE1 rabbit mAb (ab108600, Abcam, MA, USA), anti-phosphoserine/threonine/tyrosine mouse mAb (ab15556, Abcam, MA, USA), anti-Myc-Tag (9B11) mouse mAb (#2276S, Cell Signaling Technology, MA, USA) and anti-phospho-(Ser) Arg-X-Tyr/Phe-X-pSer motif rabbit Ab (#2981S, Cell Signaling Technology, MA, USA).

### Immunoprecipitation

Tissues and cells were lysed in IP lysis buffer (Beyotime, Jiangsu, China), and 500 μg samples of total protein were incubated with specific antibodies and protein A/G PLUS agarose beads (Santa Cruz, CA, USA)) overnight at 4 °C. The beads were washed with IP lysis buffer, and western blotting was then performed. The antibodies used were anti-Myc (9B11) mouse mAb (#2276S, Cell Signaling Technology, MA, USA), anti-HSPB1 (D6W5V) rabbit mAb (#95357, Cell Signaling Technology, MA, USA), anti-NPM1 rabbit pAb (10306-1-AP, Proteintech, IL, USA) and anti-HSPE1 rabbit mAb (ab108600, Abcam, MA, USA).

### Sample preparation for LC-MS/MS

Total protein was extracted from tissues using lysis buffer (PTM Bio, Zhejiang, China). Samples were sonicated at least three times on ice using an ultrasonic processor (Scientz, Zhejiang, China). To obtain peptides, samples were reduced with DTT and alkylated with iodoacetamide in the dark at room temperature, digested with trypsin, desalted using a Strata X C18 SPE column (Phenomenex, CA, USA) and vacuum-dried. Peptides were reconstituted and processed with a TMT kit/iTRAQ kit according to the manufacturer’s instructions.

### HPLC fractionation and enrichment of O-GlcNAcylated peptides and phosphorylated peptides

Peptide fractions were acquired on a Thermo Betasil C18 column (5 μm particles, 10 mm ID, 250 mm length) through high pH reversed-phase HPLC. For enrichment of O-GlcNAc-modified peptides, tryptic peptides were incubated with prewashed O-GlcNAc antibody beads (PTM-954, PTM Bio, Zhejiang, China) and incubated in NETN buffer (100 mM NaCl, 1 mM EDTA, 50 mM Tris-HCl, 0.5% NP-40 (pH 8.0)) at 4 °C with gentle shaking overnight. Immunocomplexes were washed with NETN buffer four times and were then washed with double distilled water. We used 0.1% trifluoroacetic acid to elute the bound fractions from the beads. The collected peptides were vacuum dried, followed by desalting with C18 ZipTips (Millipore, MA, USA) according to the manufacturer’s instructions. For enrichment of phospho-modified peptides, tryptic peptide mixtures were mixed with IMAC microspheres in loading buffer (50% acetonitrile/6% trifluoroacetic acid) with gentle vibration. After centrifugation, the supernatant was removed, and IMAC microspheres with bound phosphopeptides were acquired. These IMAC microspheres were washed with 50% acetonitrile/6% trifluoroacetic acid and 30% acetonitrile/0.1% trifluoroacetic acid continuously to remove nonspecifically adsorbed peptides. The phosphopeptides were then eluted from the IMAC microspheres in elution buffer with vibration. Finally, the peptides were lyophilized for LC-MS/MS analysis.

### LC-MS/MS analysis

Formic acid (0.1%, solvent A) containing the O-GlcNAc-modified peptides or phospho-modified peptides was loaded onto a reversed-phase analytical column (15 cm length, 75 μm ID). A concentration gradient was used with an increase from 6% to 23% 0.1% formic acid in 98% acetonitrile (solvent B) for 26 min; an increased from 23% to 35% for 8 min; an increase to 80% for 3 min; and maintenance at 80% for the last 3 min. The flow rate was constant at 400 nL/min on an EASY-nLC 1000 UPLC system.

Peptides were first exposed to an NSI source and were then analyzed by tandem mass spectrometry (MS/MS) in a Q Exactive^TM^ Plus (Thermo, MA, USA) coupled to a UPLC system. The specific parameters were as follows: a 2.0 kV electrospray voltage was used; the m/z scan was set from 350 to 1800; and the resolution of the Orbitrap was set as 70,000 to detect intact peptides. Next, the NCE (set to 28) and Orbitrap (resolution = 17500) were used to select peptides for MS/MS and detect the fragments. The data-dependent procedure alternated between one MS scan followed by 20 MS/MS scans with the automatic gain control set at 5E4 and a fixed first mass of 100 *m*/*z*.

### Database search

We used the MaxQuant search engine (v.1.5.2.8) to analyze the MS/MS data. The tandem mass spectra were searched against the SwissProt Human database concatenated with a reverse decoy database. The data were also searched with a common contamination database to eliminate the impact of contaminated proteins. Trypsin/P was specified as the cleavage enzyme, allowing up to two missed cleavages. The mass tolerance for precursor ions in the first search and the main search was set to 20 ppm and 5 ppm, respectively. The mass tolerance for fragment ions was set to 0.02 Da. Cysteine carbamidomethylation was specified as the fixed modification. Oxidation of methionine, acetylation of N-terminal proteins and O-GlcNAcylation of serine and threonine were specified as variable modifications. The false discovery rate for protein and peptide-to-spectrum match identification was adjusted to 1%.

### Plasmids and transfection

Myc-tagged wild-type HSPB1 and mutants, including Myc-HSPB1-T180A, Myc-HSPB1-S187G, and Myc-HSPB1-T180A/S187G, were chemically synthesized by GenePharma (Shanghai, China) and subcloned into the pEX-3 expression plasmid (GenePharma, Shanghai, China). All vectors were tested and confirmed by DNA sequencing. Cells were transfected with overexpression plasmids using ExFect Transfection Reagent (Vazyme, Jiangsu, China) in accordance with the manufacturer’s instructions. Western blotting was conducted to evaluate the transfection efficiency 48 h later.

### Cell counting Kit-8 (CCK-8) assay

Cells were seeded into 96-well plates at a density of 5000 cells per well. After treatment, 10 μl of CCK-8 reagent (Beyotime, Jiangsu, China) was added, and cells were incubated at 37 °C for 4 h. The absorbance of each well was detected at a wavelength of 450 nm by a multimode plate reader.

### Statistical analysis

All data were processed by GraphPad Prism 6 software (CA, USA) and are presented as the means ± SDs. Student’s *t* test was performed to analyze the differences between groups. In all experiments, *p* < 0.05 was considered statistically significant.

## Supplementary information

Supplementary Data S1

Supplementary Data S2

Supplementary Data S3

Supplementary Data S4

Supplementary Data S5
